# CCL2 nitration is a negative regulator of chemokine-mediated inflammation

**DOI:** 10.1038/srep44384

**Published:** 2017-03-14

**Authors:** Catriona E. Barker, Sarah Thompson, Graeme O’Boyle, Hugues Lortat-Jacob, Neil S. Sheerin, Simi Ali, John A. Kirby

**Affiliations:** 1 Applied Immunobiology and Transplantation Group, Institute of Cellular Medicine, Medical School, University of Newcastle Upon Tyne, NE2 4HH, UK; 2 Faculty of Applied Science, University of Sunderland, Sunderland, SR1 3SD, UK; 3 Institut de Biologie Structurale, UMR 5075, Univ. Grenoble Alpes, CNRS, CEA, F-38027, Grenoble, France

## Abstract

Chemokines promote leukocyte recruitment during inflammation. The oxidative burst is an important effector mechanism, this leads to the generation of reactive nitrogen species (RNS), including peroxynitrite (ONOO). The current study was performed to determine the potential for nitration to alter the chemical and biological properties of the prototypical CC chemokine, CCL2. Immunofluorescence was performed to assess the presence of RNS in kidney biopsies. Co-localisation was observed between RNS-modified tyrosine residues and the chemokine CCL2 in diseased kidneys. Nitration reduced the potential of CCL2 to stimulate monocyte migration in diffusion gradient chemotaxis assays (p < 0.05). This was consistent with a trend towards reduced affinity of the nitrated chemokine for its cognate receptor CCR2b. The nitrated chemokine was unable to induce transendothelial monocyte migration *in vitro* and failed to promote leukocyte recruitment when added to murine air pouches (p < 0.05). This could potentially be attributed to reduced glycosaminoglycan binding ability, as surface plasmon resonance spectroscopy showed that nitration reduced heparan sulphate binding by CCL2. Importantly, intravenous administration of nitrated CCL2 also inhibited the normal recruitment of leukocytes to murine air pouches filled with unmodified CCL2. Together these data suggest that nitration of CCL2 during inflammation provides a mechanism to limit and resolve acute inflammation.

The mechanisms by which ongoing inflammation is resolved are unclear. Limiting the production of pro-inflammatory factors, including chemokines, is one potential mechanism. Others include the generation of steroids, nitric oxide, adenosine, interleukin-10 and regulatory T cells^1^,^2^. A recently-described further candidate is endogenous chemical modification of existing cytokines leading to alteration of their biological properties.

The infiltration of immune cells able to perform oxidative burst is a major and recurring cause of tissue injury during inflammation. Macrophages and neutrophils produce nitric oxide and the superoxide anion by inducible NO synthase and NADPH oxidase respectively, which react to form peroxynitrite. Reactive oxygen species, for example H_2_O_2,_ are well known to be associated with inflammation^3^. However, fewer studies have explored the contribution of RNS such as peroxynitrite^4^. Peroxynitrite is a major oxidant in pathological conditions associated with oxidative stress including diabetes^5^, organ transplant^6^ and cancer^7^. Peroxynitrite can spontaneously nitrate aromatic amino acids including tyrosine and tryptophan, and it also oxidises the thiol group of methionine and cysteine to sulfoxide. The short half-life of peroxynitrite prevents its detection *in vivo*, but the biomarker 3-nitrotyrosine is indicative of its presence. It is known that nitration can modify protein functions^8^. For example, peroxynitrite can alter the function of transcription factors such as NFκB resulting in, altered cell adhesion molecule expression^9^. Oxidative stress can alter other transcription factors such as HIF-1α and p53 further developing the stress response, and can also increase mitochondrial permeability allowing signalling molecules to transition both to and from the nucleus^10^.

Chemokines, are small (8–14 kDa) chemoattractant cytokines secreted by many cell types. Chemokines can be regulated at the transcriptional and translational levels by oxidative stress. Some preformed chemokines are stored in endothelial cells, in secretory granules including Weibel-Palade bodies allowing a rapid response to insult^11^. Once produced, the functions of chemokines are also closely regulated by post-translational modification^12^, receptor expression and GAG binding^13^. Chemokines typically bind and signal through G-protein coupled receptors but decoy, or atypical, receptors can also bind chemokines, generally with anti-inflammatory effects^14^. In order to form the chemokine gradients needed for *in vivo* function, chemokines bind to GAGs such as heparan sulphate^15^,^16^. Endothelial expression of these cell surface GAGs increases during the stresses induced by transplantation, resulting in increased endothelial potential to bind and present chemokines^17^.

Chemokines are well known targets for post-translational modification, with enzymatic processing altering their biology^6^,^18^,^19^. For example, matrix metalloproteinases are released by stressed cells and can cleave chemokines^20^, whilst peptidylarginine deiminases can inactivate certain chemokines by citrullination^21^. Several chemokines have also been found to be targets of peroxynitrite modification, including CCL2^8^,^22^,^23^ and CCL5^8^. Generally it is thought that modification of proinflammatory cytokines by peroxynitrite abrogates function, but this is dependent on numerous factors, including cell type, anti-oxidant levels (scavenger glutathione) etc. A recent study showed that peroxynitrite-treated CCL2 lost its ability to recruit CD8+ T cells, but the recruitment of myeloid-derived suppressor cells was unaltered^23^. No mechanisms of action have been defined. Molon *et al*.^23^ reported that nitrated CCL2 cannot be detected by commercially available antibodies as the modification causes epitope loss. This has important implications for studies using antibody based techniques to study chemokine levels in inflammatory environments.

The current study was designed to explore the potential of nitrated chemokines to regulate inflammation. CCL2 was chosen as a prototypical chemokine as it is produced by various cell types in response to diverse stimuli including oxidative stress. Initial experiments were performed to validate the potential expression of nitrated CCL2 in inflamed human renal tissue biopsies. We then used several models to demonstrate that nitration reduced the potential of CCL2 to induce inflammation. Importantly, we also showed that nitrated CCL2 was able to inhibit the activity of unmodified CCL2 *in vivo*. A final experiment demonstrated that nitration removed the potential of CCL2 to bind heparan sulphate suggesting a mechanism for the anti-inflammatory activity of this inflammation-modified chemokine.

## Results

### Protein nitration in ischaemia–reperfusion injury

**Figure 1:**
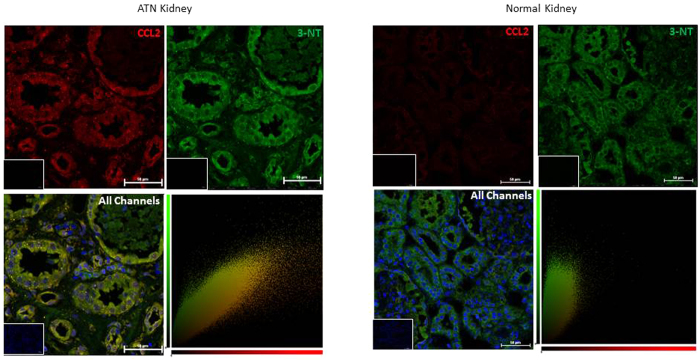
Protein nitration in ischaemia–reperfusion injury. Kidney sections from patients diagnosed with ATN as a result of ischaemia-reperfusion injury sustained during kidney transplant were stained by immunofluorescence for 3-nitrotyrosine and CCL2. 3-nitrotyrosine staining was developed with a FITC conjugated secondary (green), CCL2 was developed with a Dylight-550 conjugated secondary (red), and nuclei were counterstained with DAPI (blue). Four patient samples, and six normal kidney samples were tested and representative images are shown. Original magnification x20, taken on Nikon A1 Confocal microscope, with co-localization analysis performed using Volocity software. Bars, 50 μm. Insert - no primary antibody control.

In order to examine the effects of oxidative stress *in vivo*, we used biopsy sections taken from patients who had been diagnosed with acute tubular necrosis (ATN) caused by ischaemia reperfusion injury (IRI) sustained during kidney transplantation. This was compared to staining in kidney sections from the unaffected pole of tumour nephrectomies. 3-nitrotyrosine appeared to be present constitutively, particularly in the tubules, in both normal and ATN kidneys (Fig. 1); a staining pattern which has been previously documented^24^. However, it is clear that expression of CCL2 within the tubules increases greatly after IRI in comparison to the levels observed in normal kidneys. The staining patterns for CCL2 and 3-nitrotyrosine co-localize largely in the tubules of the injured kidney (confirmed by the yellow pixels indicating co-localization in the cytofluorograms) suggesting the presence of NO_2_-CCL2 at this site. Presence of increased amount of nitrated chemokine was also detected in rejecting cardiac transplant biopsy (Supplementary Figure 1).

Chemokines including CCL2 are produced during IRI. Furthermore, RNS modulate inflammation by post-translational modification of chemokines. In order to examine the role of nitrated CCL2 in inflammation we exposed CCL2 to peroxynitrite for 10 minutes and the presence of modification was determined by tryptic digest and tandem mass spectrometry coupled to liquid chromatography (LC-MS/MS). Both tyrosines (Y13 and Y28) and tryptophan (W59) were reproducibly modified by nitration. Methionine oxidation was also found but is a common artefact during mass spectrometry which cannot be attributed solely to peroxynitrite (Supplementary Figure 2).

### Effect of nitration on biological activity of CCL2

**Figure 2:**
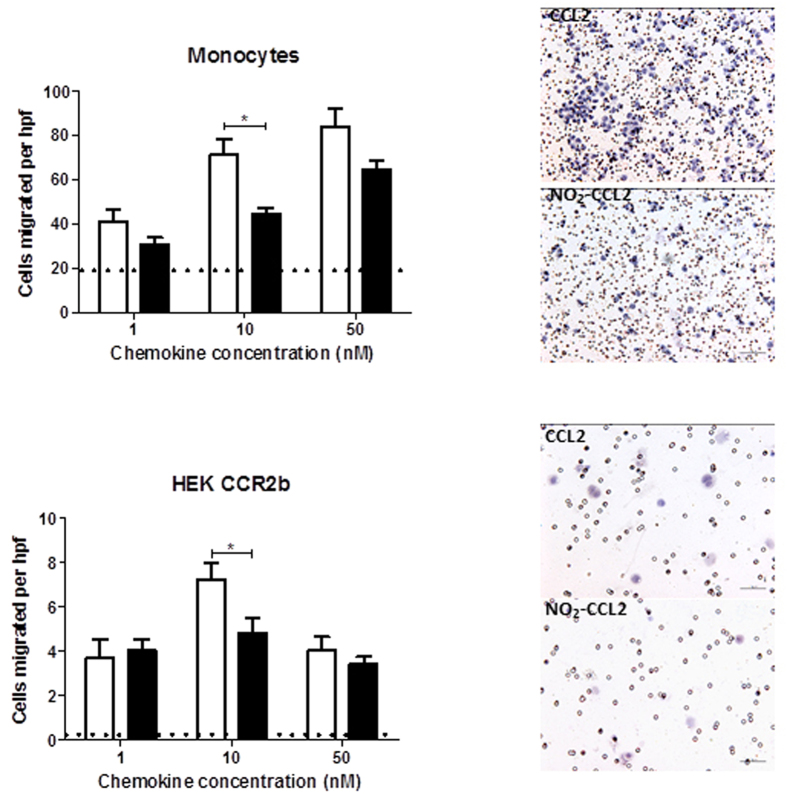
Diffusion gradient chemotaxis comparing the ability of WT-CCL2 and NO_2_-CCL2 to elicit migration. Migrated monocytes and HEK-CCR2b adhered to the underside of the filter and 5 high power fields (hpf; 400×) counted per filter. Data shows nitration decreases the chemotactic ability of CCL2 for monocyte migration and at least some of this is through CCR2b. White bars - CCL2, black bars - NO_2_-CCL2. No chemokine control displayed by dotted lines. Representative data of three independent experiments, performed in triplicate. Data is presented as mean ± SEM, statistical analysis by Student’s t-test. Images shown are examples of a high power field (HPF) taken at 20× magnification from the 10 nm filters.

In order to examine the biological properties of NO_2_-CCL2, chemotaxis assays were carried out. Figure 2 shows CCL2 nitration decreases its ability to recruit monocytes in a dose dependent manner using diffusion gradient chemotaxis assays. Furthermore, to determine whether nitration affected the interaction between CCL2 and its cognate receptor CCR2b, the effect of CCL2 nitration on chemotaxis of HEK-CCR2b transfectants was assessed (signalling via a single receptor). Migration of HEK-CCR2b transfectants was significantly reduced at 10 nM concentration of NO_2_-CCL2 compared with WT-CCL2, indicating that nitration alters the biological activity of CCL2. As no significant difference was observed at 50 nM, this suggests that the reduction in migration in response to 10 nM NO_2_-CCL2 is due to nitration reducing the chemokine activity; an affect that can be countered by saturating the system with a higher concentration of nitrated chemokine.

### Effect of nitration on binding of CCL2 to its receptor CCR2

**Figure 3:**
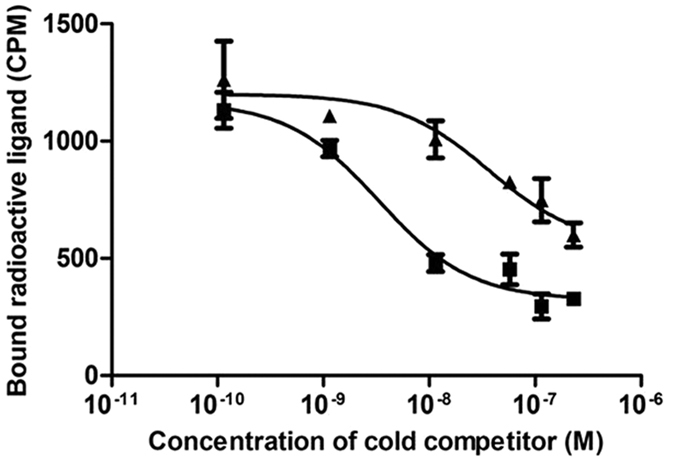
Radioligand binding assays. Results from a representative heterologous cold competition-binding assay conducted using HEK-293 cells transfected to stably express CCR2b, ^125^I-labeled CCL2 as the radiolabelled ligand, and a variable concentration of either unlabelled CCL2 (▪) or NO_2_-CCL2 (▴) as competitors. Results shows nitration decreases receptor binding (IC_50_ CCL2 = 3 nM, NO_2_-CCL2 = 36 nM).

To determine if the decrease in recruitment of HEK-CCR2b in response to NO_2_-CCL2 compared to CCL2 was due to decrease in CCR2b binding, a series of radioligand binding assays were carried out. Representative homologous cold-ligand competition curves for ^125^I-CCL2 are shown in Fig. 3. The IC_50_ value for WT-CCL2 and NO_2_-CCL2 was found to be 3.71 ± 1.138 and 21.2 ± 8.2 nM respectively (mean ± S.D., n = 3). Although this difference did not reach statistical significance, the trend suggests that nitration reduced the affinity of CCL2 for CCR2 around 6-fold.

### Transendothelial chemotaxis

**Figure 4:**
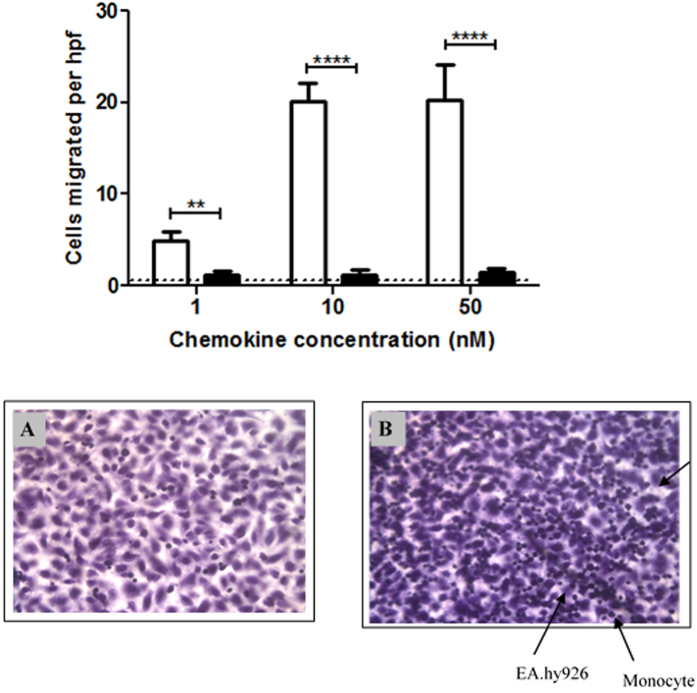
Chemotaxis comparing the ability of WT-CCL2 and NO_2_-CCL2 to elicit migration of monocytes across an EAhy.926 monolayer. Monocytes adhered to the underside of the filter and 5 hpf were counted per filter. Data shows nitration abrogates transendothelial migration in response to CCL2. White bars - CCL2, black bars - NO_2_-CCL2. No chemokine control displayed by dotted lines. Methanol-fixed filters (A) 10nM NO_2_-CCL2, B) 10nM wild type CCL2) stained with heamotoxylin, monocytes stain more intensely than EAhy.926 s resulting in monocytes appearing as dark spots on a background of EAhy.926 s stained lighter purple. Cells which were sharply in focus on the lower surface of the filter membrane, but not on the upper surface were counted as migrant. Representative data of three independent experiments, performed in triplicate. Statistical analysis by Student’s t-test.

A further series of experiments was performed to compare the potential of WT and NO_2_-CCL2 to stimulate the migration of monocytes across endothelium. In these assays, endothelial cells were cultured to confluency on the upper surface of a porous filter. Both the WT or NO_2_-CCL2 was then added to the basal compartment and mononuclear cells were introduced above the apical surface of the model endothelium. In contrast to the results for trans-filter migration, it was found that the NO_2_-CCL2 stimulated no increase in transendothelial cell migration when used at 1, 10 and 50 nM concentrations (p < 0.05; Fig. 4). However, WT-CCL2 stimulated significantly increased leukocyte migration at each of the tested concentrations (all p < 0.001).

### Effect of nitration on Glycosaminoglycan (GAG) interaction

**Figure 5:**
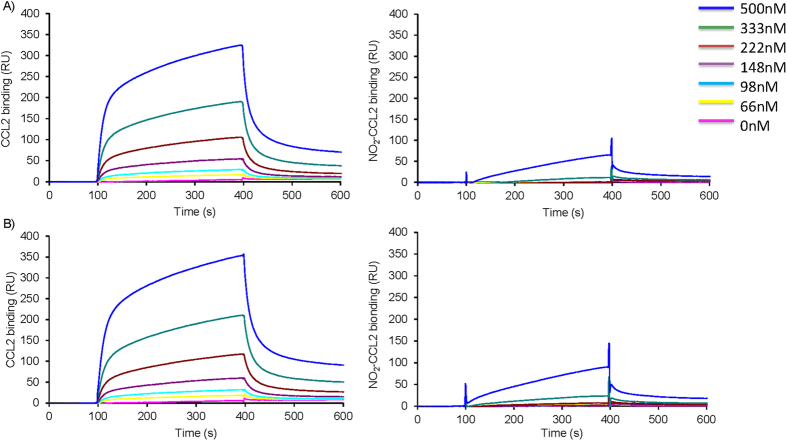
Surface plasmon resonance assessing the effect of nitration on CCL2- heparan sulphate interaction. Chemokines at a range of concentrations (66–500 nM) were injected over sensor chip-immobilised **A**) heparan sulphate or **B**) heparin, WT-CCL2 left and NO2-CCL2 right, for 5 min, after which running buffer was injected, and the response in resonance units (RU) was recorded as a function of time. Biacore sensograms are representative of two experiments.

To assess the impact of nitration on chemokine GAG binding WT-CCL2 and NO_2_-CCL2 were assessed in solid phase heparin binding assays. The NO_2_-CCL2 did not significantly bind to immobilised heparin. This result was confirmed by Surface Plasmon Resonance (SPR) measurements in which reducing end biotinylated heparan sulphate (or heparin) was captured on top of a streptavidin sensorchip. This system mimicked cell membrane-anchored proteoglycans and SPR spectroscopy was used to measure changes in the refractive index caused by the interaction that occurred when a range of concentrations of WT or NO_2_-CCL2 were flowed across the immobilized GAG sensorchips. The resulting sensorgrams (Fig. 5) demonstrated that WT-CCL2 can interact with heparan sulphate, whereas no specific binding could be detected for NO_2_-CCL2 at concentrations up to 500 nM. Similar results were observed for interactions with heparin.

### Investigation of the role of nitration in chemokine-mediated inflammation

**Figure 6:**
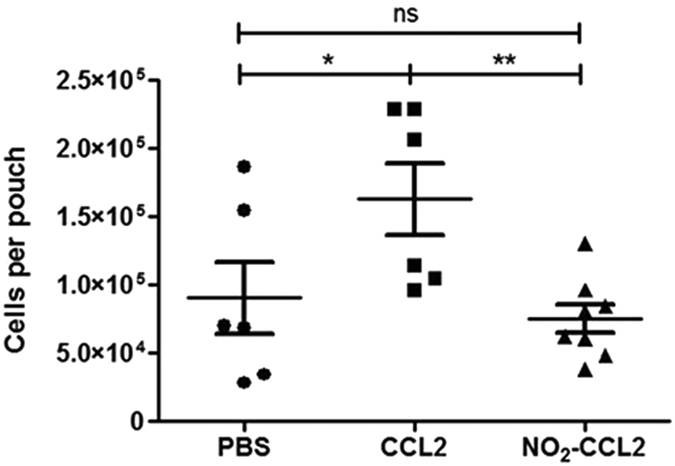
Functional effect of CCL2 nitration on cell recruitment *in vivo* using a murine air pouch model of chemotaxis. Total cell migration into air pouches 18 hr after intrapouch administration of 10 μg WT-CCL2, NO_2_-CCL2 or PBS only control was determined. Cells were counted by haemocytometer. Statistical analysis by ANOVA with Bonferroni post-test. Each symbol represents an animal.

The stable air pouches formed on the back of mice develop a lining which resembles the synovial membrane^25^. Introduction of 10 μg of WT-CCL2 to air pouches induced significant recruitment of leukocytes within 18 h. Recruitment in response to CCL2 was significantly higher than to either PBS or NO_2_-CCL2, with migration in response to NO_2_-CCL2 and PBS not significantly different (Fig. 6). Interestingly, the mean recruitment of cells in response to NO_2_-CCL2 is lower than that of PBS group, although this did not reach significance.

**Figure 7:**
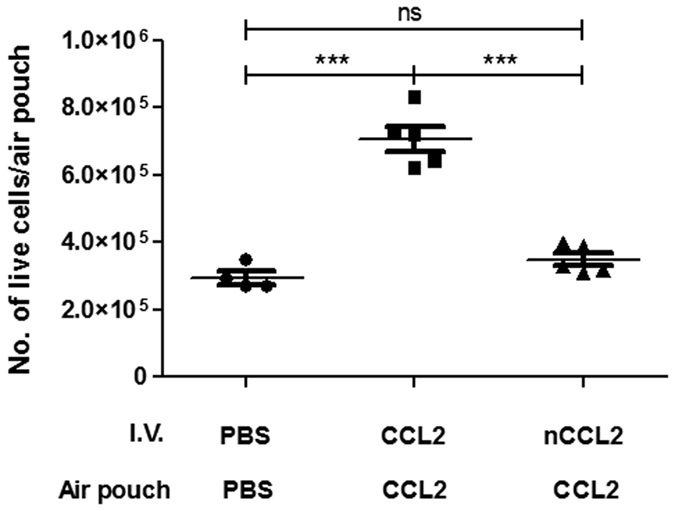
NO_2_-CCL2 can antagonise the effects of CCL2 *in vivo*. Total cell migration into murine air pouches was determined 18 hr after intrapouch administration of 10 μg CCL2 or PBS only control and I.V. administration of 20 μg CCL2, NO_2_-CCL2 or PBS only control. Cells were counted by haemocytometer. Statistical analysis by ANOVA with Bonferroni post-test. Each symbol represents an animal.

A further series of experiments was performed to assess the effect of intravenous administration of NO_2_-CCL2 on the intra-air pouch inflammatory response. The negative control group, with intravenous PBS and intrapouch PBS, showed levels of infiltrating leukocytes that were similar to those in earlier experiments (Fig. 7). Interestingly, intravenous administration of NO_2_-CCL2 to animals with WT-CCL2 in their air pouches resulted in a significant decrease in the number of cells recruited to the pouch compared with groups that received WT-CCL2 intravenously and WT-CCL2 in pouch (P < 0.001). These data suggest that systemic administration of NO_2_-CCL2 is able to inhibit localized, CCL2-mediated inflammation within the air pouch.

## Discussion

During inflammation leukocytes migrate to the damaged site to neutralise the induction of chemotactic stimuli. It is generally believed that the removal of stimulus will stop any further pro-inflammatory mediator synthesis (e.g. chemokine, adhesion molecules) allowing resolution of inflammation. However, several anti-inflammatory agents including nitric oxide, IL-10^26^ and endogenous negative regulators of leukocyte recruitment such as pentraxin-3 and endothelium-derived developmental endothelial locus-1 (Del-1) have been identified^27^. These observations have challenged the concept that the resolution of inflammation is simply a passive process.

Previous studies have shown that modification of proinflammatory cytokines and chemokines by peroxynitrite can alter their function^8^,^22^,^23^. Peroxynitrite can selectively oxidise and nitrate several residues, including the oxidation of histidine and the nitration of tyrosine and tryptophan. Nitration of tyrosine results in the formation of 3-nitrotyrosine. This gives the modified residue a 45 Da mass increase but the overall charge remains unaltered. 3-nitrotyrosine is a marker of nitro-oxidative stress and the presence of peroxynitrite is seen in many pathological conditions, including diabetes and transplantation^4^. Antibodies have been raised against 3-nitrotyrosine and are used to identify nitrated proteins and the presence of peroxynitrite or related species^23^. Our data demonstrates the use of such antibodies to show co-localisation of 3-nitrotyrosine and CCL2 in kidney samples from patients with ATN. Chemokine nitration has previously been identified *in vivo* for CCL2 by immunohistochemistry and ELISA^23^.

NO_2_-CCL2 showed reduced directional leukocyte migration in a chemokine concentration gradient produced by free solute diffusion (trans-filter). This reduced potential was seen both with T cells (data not shown) and monocytes; however cell recruitment in response to NO_2_-CCL2 remained significantly higher than the background. Furthermore, in this study it has been demonstrated that NO_2_-CCL2 can interact with specific chemokine receptors. Although nitration caused a reduction of affinity by around 6-fold, previous studies have shown that this level of reduction in affinity is not sufficient to abrogate the function of chemokine^28^.

It is unlikely that a stable solute concentration gradient could occur *in vivo* for periods of time required for tissue inflammation. We further examined the ability of nitrated chemokine to support transendothelial migration, a more physiologically relevant technique requiring apical presentation of chemokine was carried out. It was found that unlike WT-CCL2, the NO_2_-CCL2 was not capable of inducing transendothelial leukocyte migration *in vitro*. A similar failure of cell migration has been reported previously for leukocyte transmigration assays performed using non-GAG binding chemokines^15^,^29^,^30^.

The residues nitrated in CCL2 were identified but exactly which modified residues are responsible for modulation in function is not known. Tyr13 is one of the residues modified and has previously been shown to be crucial for CCL2 function^31^,^32^,^33^. It is involved in receptor binding and signalling and so modification of this amino acid could explain the decrease in receptor affinity and diffusion gradient chemotaxis. Our group and others have shown that the interaction between chemokines and GAGs is of crucial importance for inflammatory migration of leukocytes through monolayers of endothelial cells^15^,^30^,^34^. GAG interaction assessed by Biacore showed that NO_2_-CCL2 is unable to bind both heparin and heparan sulphate. CCL2 binding was achieved but no kinetic models could be fitted to the data. This is most probably due to complexities of CCL2-GAG interactions. CCL2 can be present as a monomer, dimer and large oligomers and both heparan sulphate and heparin, being polymeric and heterogeneous in sequence, can display several binding sites with distinct affinities. This suggests that although NO_2_-CCL2 binds to CCR2b, albeit with reduced affinity its inability to support trans-endothelial migration is most likely due to reduction in GAG binding.

The potential mechanism by which nitration reduces the interaction with GAGs could be by disruption of dimerization. Previous studies have shown that dimerisation of CCL2 is crucial for GAG binding and P8A and Y13A mutants are obligate monomers^35^,^36^. Supplementary Figure 3 highlights the position of Tyr13 along dimer interface. If nitration of Tyr13 affects the ability of CCL2 to dimerise either by altering hydrogen bonding or conformation of the protein then GAG binding could be affected. However, the loss in GAG binding in the Y13A mutant was not as profound as in NO_2_-CCL2. Dimerisation is therefore unlikely to be the sole explanation for decreased function. Another explanation for the substantial loss of GAG interaction could potentially be due to unfolding of the protein. However, our receptor binding data and the ability of NO_2_-CCL2 to support diffusion gradient chemotaxis (albeit at reduced rate) suggests that the N-terminal region is structurally intact.

As expected, the series of *in vivo* experiments showed that injection of WT-CCL2 resulted in recruitment of leukocytes into murine air pouches. In contrast, injection of NO_2_-CCL2 produced no significant influx of inflammatory leukocytes. The *in vivo* data is consistent with the transendothelial chemotaxis assays performed *in vitro*, and suggests that the presentation of GAG-bound chemokine is also required to promote the physiological leukocyte migration which leads to inflammation.

A further series of experiments was performed to assess the potential of systemic administration of NO_2_-CCL2 to modulate the infiltration by mononuclear leukocytes of WT-CCL2 filled air pouches. Importantly, it was found that i.v. administration of WT-CCL2 enhanced infiltration of the air pouches, while administration of NO_2_-CCL2 effectively prevented the recruitment of mononuclear leukocytes to air pouches. As seen with other non-GAG binding chemokines it is possible that the NO_2_-CCL2 exists in circulation for a longer period of time compared with WT-chemokines which are sequestered by anionic GAGs^15^. Chronic exposure to NO_2_-CCL2 in the circulation can lead to reduced leukocyte-surface expression of CCR2, reduced chemotactic response of these cells to WT-CCL2, and inhibit normal chemokine-mediated induction of adhesion.

The majority of studies assessing the involvement of chemokine in disease assume the chemokine to be fully functional. Not only is this over simplification, especially *in vivo* but may be an important confounding factor for the failure of chemokine based drug trials. Most of the current detection methods do not differentiate between different forms of chemokines. Further, analysis of the role of chemokines in disease is necessary and may explain certain paradoxes found between clinical outcome and chemokine levels^37^.

There is increasing recognition that although targeting infiltrating immune cells can control the inflammatory response it does not lead to permanent resolution of inflammation. Resolution therapies can act differently by being for example receptor agonists which switch on protective mechanisms that lead to return of homeostasis. At present no drugs have been specifically designed to activate pro-resolving pathways. However, there are drugs which mediate their affect partly by inducing the synthesis of endogenous anti-inflammatory mediators. For example methotrexate and FK506 are thought to increase the synthesis of adenosine which acts through A2A adenosine receptor to control leukocyte trafficking^38^.

The generation of non-GAG binding NO_2_-chemokines during episodes of inflammation-associated oxidative stress suggests a mechanism to limit further leukocyte recruitment leading to the resolution of inflammation. Further development of this understanding might suggest improved methods for therapeutic anti-inflammatory therapy following organ transplantation.

## Materials and Methods

All tissue culture work was carried out in accordance with the ‘Safe Working with Biological Hazards’, ‘Safe Working with Chemicals in the Laboratory’ and ‘A Basic Guide for Radiation Workers’ publications and the University of Newcastle Upon Tyne Safety Policy.

Human tissue access was approved by Research Ethics Committee approval, Ref. -11/NE/0352. Informed consent was obtained from all subjects. All animal procedures were approved by the Home Office UK and conducted in accordance with the requirements of the UK Animals (Scientific Procedures) Act 1986 (Home Office License PPL60/3772).

### Chemokine nitration

Chemokine nitration was achieved by adding peroxynitrite (Cayman Chemical) at a final concentration of 1 mM, to 1 μM CCL2 in dH_2_O and incubating at 37 °C for up to 10 min. Chemokine was freshly nitrated for each experiment and all chemokines were purchased from Almac, UK.

The exact residues modified by peroxynitrite were identified by mass spectrometry. Tryptic digestion was performed prior to LC-MS/MS and the data were analysed using Mascot (Matrix Science) as described earlier^39^.

### Immunofluorescence

Formalin-fixed paraffin-embedded (FFPE) tissue sections from patient biopsies diagnosed with ATN, 1 week post-kidney transplant were dewaxed (Research Ethics Committee approval, Ref. -11/NE/0352). Antigen retrieval was carried out using citrate buffer (pH6) for 2 mins. Sections were blocked for 30 mins with 20% normal goat serum. They were stained sequentially, with primary antibody overnight at 4 °C and secondary for 2 hours at room temperature. Anti-nitrotyrosine polyclonal (1/500, Millipore) was detected using a FITC conjugated secondary antibody (1/300, Sigma), and anti-CCL2 polyclonal (1/500, Abcam) was detected using a Dylight-550 conjugated secondary antibody (1/100, Immunoreagents). Slides were then incubated in 0.1% Sudan Black B in 70% Ethanol for 20 minutes at room temperature, washed in PBS, and mounted using Vectashield mounting medium (Vector Labs). Co-localization analysis was performed, and cytofluorograms generated, using Volocity software (Perkin Elmer).

### Cell isolation and culture

Peripheral blood mononuclear cells (PBMC) were isolated from whole blood taken from healthy volunteers. Ethical approval to obtain blood from healthy volunteers was granted by the Research Ethics Committee (12/NE/0121). PBMC were isolated from heparinised blood using Lympholyte-H (Cedarlane Laboratories) as per manufacturer’s instructions. Cells were serum starved for 1 hr prior to use in assays.

HEK-CCR2b cells^40^ were cultured in complete DMEM with 800 μg/ml G418 (Calbiochem). The THP-1 monocytic cell line (ATCC TIB-202) was cultured in complete RPMI-1640.

### Chemotaxis assays

Diffusion gradient chemotaxis assays were performed in a transwell system in 24 well companion plates (BD Falcon) as described previously^34^. For monocyte chemotaxis, 5 × 10^5^ cells were placed in the upper well of a 3 μm cell culture insert above 0–50 nM CCL2 or NO_2_-CCL2 in 0.1% BSA/RPMI. The assay was incubated at 37 °C for 90 min. Migrated monocytes adhered to the underside of the filter were fixed in methanol, stained with haematoxylin, and 5 high power fields (x400) were counted per filter.

For HEK-CCR2b chemotaxis, fibronectin coated 8 μm inserts were used. The undersides of filters were coated with 80 μl 2.5 μg/ml fibronectin (Sigma) for 30 min. 2 × 10^5^ cells were placed in the upper well and allowed to migrate towards a range of chemokine concentrations for 6 hr at 37 °C.

*In vitro*, transendothelial chemotaxis assays were performed as above (monocytes) except 72 hr prior to the assay, 5 × 10^4^ EA.hy-926 cells were cultured in 0.5 ml complete RPMI in cell culture inserts^29^. All assays were performed in triplicate.

### Chemokine receptor binding assays

2 × 10^5^ HEK-CCR2b per tube were resuspended in 30 μl binding buffer (1 mM CaCl_2_, 5 mM MgCl_2_, 50 mM HEPES, 0.5% BSA, pH7.2). 100 μl CCL2 or NO_2_-CCL2 in binding buffer was then added at a final concentration of 0.1–650 nM, along with a tracer amount of [^125^I]-CCL2 (Perkin Elmer) in 20 μl binding buffer. Tubes were incubated on ice with agitation for 2 hr. Cells were then washed twice using wash buffer (binding buffer with 0.5 M NaCl) before resuspending in 1 ml dH_2_O. A negative control with unlabelled chemokine was also performed. Counts per minute (CPM) were measured using a gamma counter (Perkin Elmer) and background radiation readings subtracted from results. Experiments were performed three times in triplicate and IC_50_ values calculated using ‘one site competition analysis’ using Prism 5.0 software (GraphPad, San Diego, CA, USA).

### Heparan sulphate binding assays

Surface plasmon resonance was performed using a BIAcore 3000 as described previously^41^,^42^. Briefly, heparin or heparan sulphate was biotinylated at the reducing end and immobilised to a CM4 sensor chip (GE Healthcare). The chip surface was activated with 50 μl 0.2 M 1-ethyl-3-(3-dimethylaminopropyl)-carbodiimide and 50 μl 0.05M N-hydroxysuccinimide before injection of 50 μl of streptavidin (0.2 mg/ml in 10 mM acetate buffer, pH 4.2). Remaining activated groups were blocked with a 5 min injection of 1 M ethanolamine HCl, pH 8.5. Biotinylated heparan sulphate was immobilised by injecting 5 μl of 50 μg/ml HS in Hanks’ Balanced Salts with 0.3 M NaCl at a flow rate of 5 μl/min and injections repeated until a resonance unit (RU) increase of 200 reached after which the surface was then washed with 2 M NaCl. For binding assays, a range of chemokine concentrations (0–500 nM) were passed across the chip at 25 μl/min for 5 mins followed by a 500 sec dissociation phase and a 2 min injection of 1 M NaCl to regenerate the sensor surface. RU from a flow cell coated with streptavidin only was subtracted from the results from GAG coated flow cells and analysis was performed using BIAevaluation.

### Cell recruitment to murine air pouches

Eight-week-old female BALB/c mice (Charles River, UK) were used for generation of air pouches as described previously^25^. Studies were performed in accordance with the United Kingdom Animals (Scientific Procedures) Act 1986 (Home Office License PPL60/3772). Briefly, air pouches were generated by injecting 3 ml of sterile air subcutaneously into the back of each animal, followed by 1 ml of air on day 2, 4, and 5, creating stable fluid-filled pouches. On day 6, each pouch was injected with 1 ml PBS containing either 10 μg CCL2 or NO_2_-CCL2. Age and sex-matched control mice were injected with PBS alone.

After 18 hr, recruited cells were recovered by gently lavaging the pouch twice with 0.75 ml of PBS. The exudates were centrifuged at 500× g for 5 min and the cells resuspended in PBS for analysis by haemocytometer and flowcytometry. An example of the gating used during flow cytometric analysis is shown in Supplementary Figure 4. In further experiments air pouches were generated as above and PBS ± 20 μg CCL2 was injected into the tail vein immediately before injection of chemokine into the pouch.

### Statistical analyses

All results are expressed as means ± SEM of replicate samples. The significance of changes was assessed by the application of an ANOVA with Bonferroni post-test or two-tailed Student’s t-test. All data were analysed using Prism 5.0 software.

## Additional Information

**How to cite this article**: Barker, C. E. *et al*. CCL2 nitration is a negative regulator of chemokine- mediated inflammation. *Sci. Rep.* **7**, 44384; doi: 10.1038/srep44384 (2017).

**Publisher's note:** Springer Nature remains neutral with regard to jurisdictional claims in published maps and institutional affiliations.

## Supplementary Material

Supplementary Figures 1,2,3,4
