# Supplementary material for: CCL2 nitration is a negative regulator of chemokine-mediated inflammation
Source: Sci Rep. 2017 Mar 14;7:44384. doi: 10.1038/srep44384 (PMC5349559; doi:10.1038/srep44384)
Supplement: Supplementary Figures 1,2,3,4 [file srep44384-s1.pdf]

CCL2 nitration is a negative regulator of chemokine-mediated inflammation

Catriona E Barker, Sarah Thompson, Graeme O'Boyle, Hugues Lortat-Jacob, Neil S Sheerin, Simi Ali* and John A Kirby

CCL2 nitration is a negative regulator of chemokine-mediated inflammation following transplantation

Catriona E Barker, Sarah Thompson, Graeme O'Boyle, Hugues Lortat-Jacob, Neil S Sheerin, Simi Ali* and John A Kirby

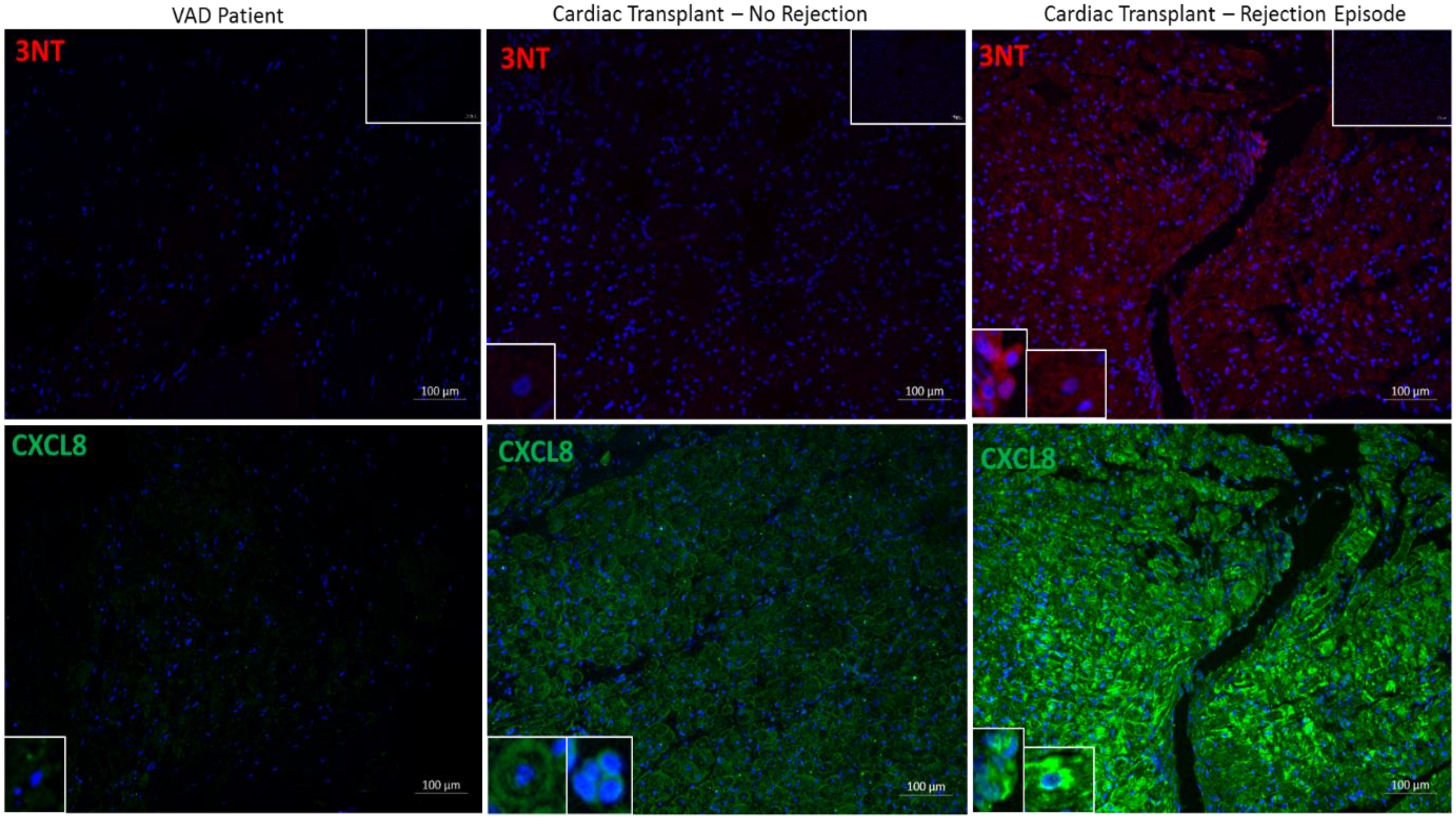

Supplementary Figure 1: Staining for 3-nitrotyrosine and CXCL8 in patient biopsies taken at the time of VAD implantation, or one month post-heart transplant with/without rejection. Markers were singly stained and detected using a Dylight550 conjugated secondary antibody pseudocoloured red (3-nitrotyrosine) or green (CXCL8), no primary controls are indented (top right). Images are taken at 10x magnification and are representative of n=5 (VAD patients), n=4 (cardiac transplant – no rejection) and n=1 (cardiac transplant – rejection episode).

CCL2 nitration is a negative regulator of chemokine-mediated inflammation following transplantation

Catriona E Barker, Sarah Thompson, Graeme O'Boyle, Hugues Lortat-Jacob, Neil S Sheerin, Simi Ali* and John A Kirby

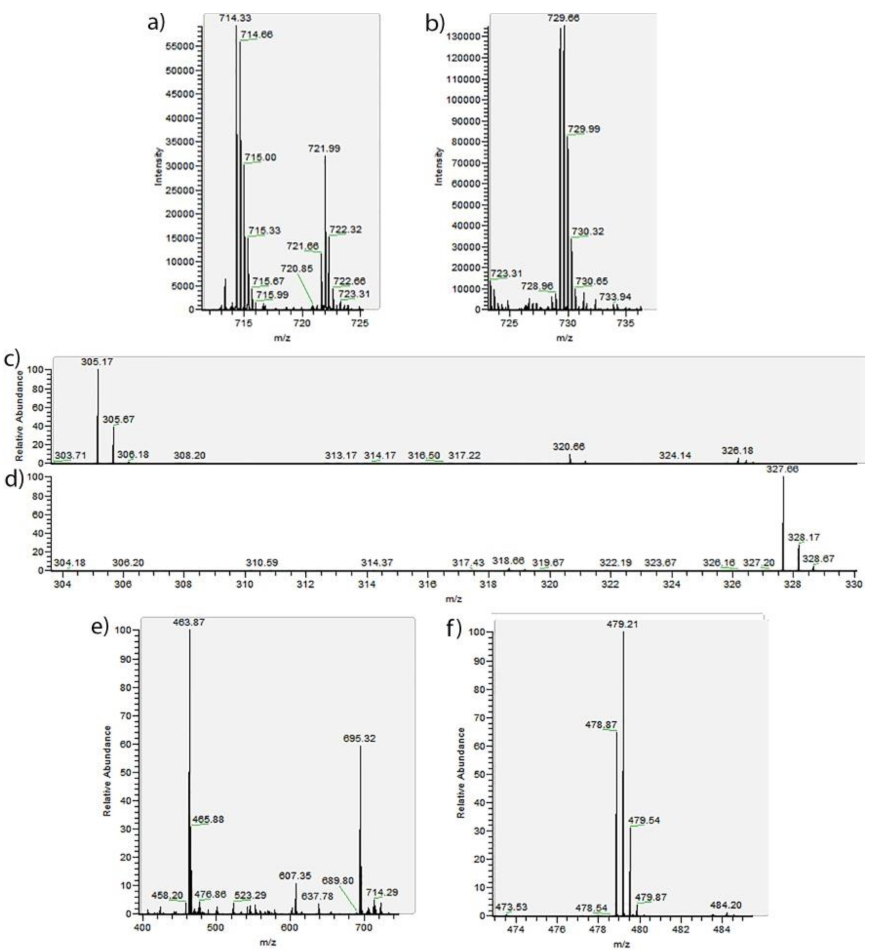

Supplementary Figure 2: MS/MS spectra of peroxynitrite incubated CCL2. Peroxynitrite modified CCL2 was analysed by LC-MS/MS following trypsin digest to establish which residues were modified. Shown is the peptide containing Tyr13 (-.QPDAINAPVTCCYNFTNR.K). a) unmodified (714.3) b) tyrosine nitration (729.6). n=2 . C) Shown is the peptide containing Tyr28 (R.LASYR.R), unmodified (305.17), d) tyrosine nitration (327.66). n=2. e) Shown is the peptide containing Trp59 (K.WVQDSMDHLDK.Q), methionine oxidation (463.87 and 695.3; can occur during MS), f) methionine oxidation with tryptophan nitration (478.87). n=2

CCL2 nitration is a negative regulator of chemokine-mediated inflammation following transplantation

Catriona E Barker, Sarah Thompson, Graeme O'Boyle, Hugues Lortat-Jacob, Neil S Sheerin, Simi Ali* and John A Kirby

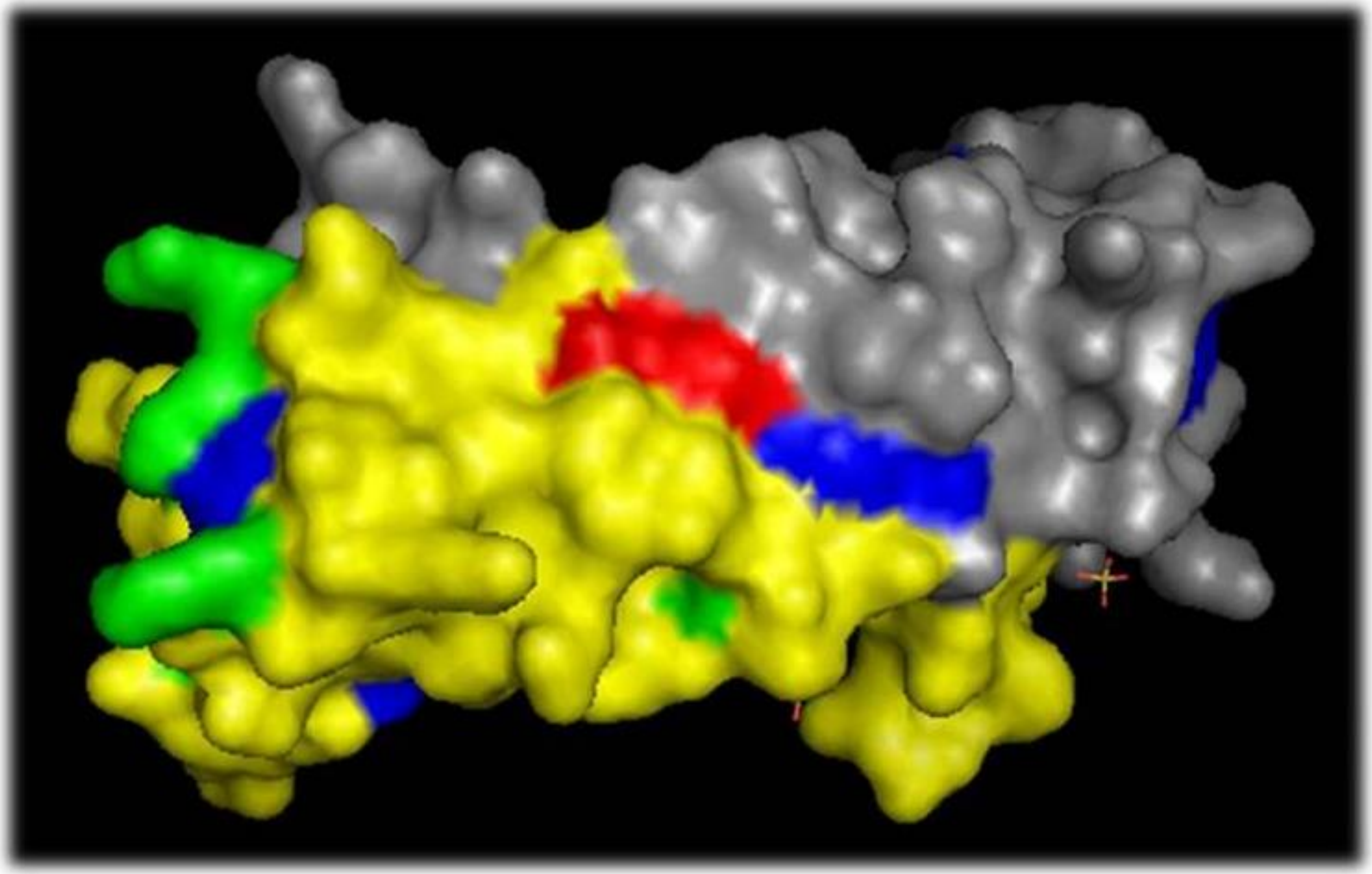

Supplementary Figure 3: CCL2 dimer structure: Cartoon of a CCL2 dimer with potential nitration sites highlighted. Tyr13 from both monomers can be seen along the dimer interface. Monomers yellow and grey, GAG binding sites – green, nitration sites - blue and red.

CCL2 nitration is a negative regulator of chemokine-mediated inflammation following transplantation

Catriona E Barker, Sarah Thompson, Graeme O'Boyle, Hugues Lortat-Jacob, Neil S Sheerin, Simi Ali* and John A Kirby

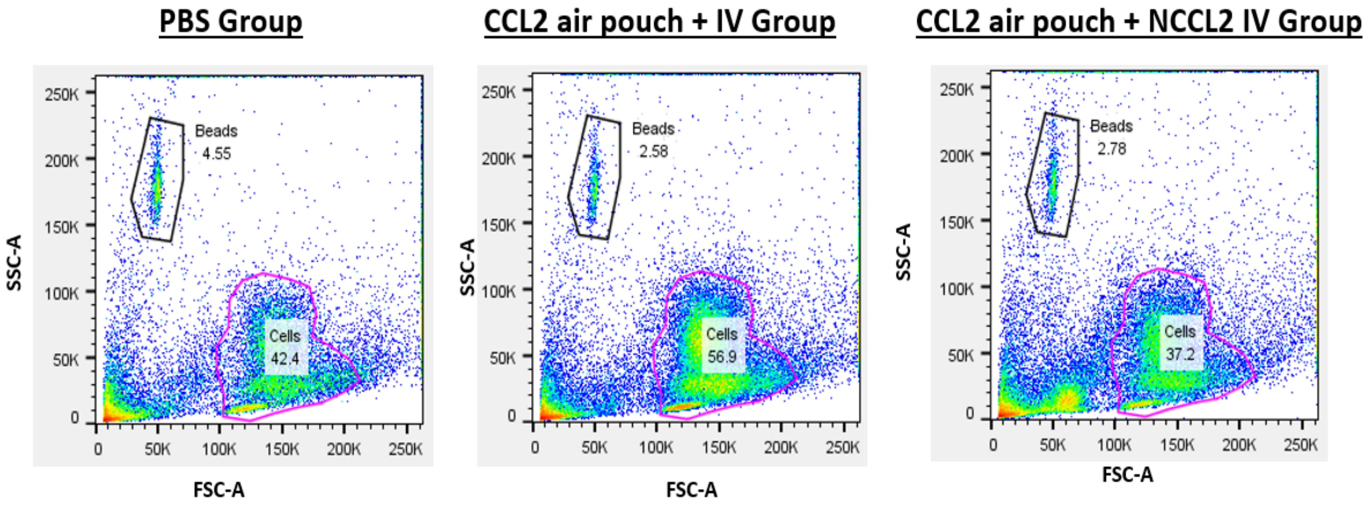

Supplementary Figure 4: An example of gating using one sample from each treatment group, showing distinct populations for beads and cells. Raw data from these populations was then used to calculate total cells (using bead manufacturer supplied equation). CCL2 = wild type CCL2, NCCL2 = nitrated CCL2.
